# ALS Patients Exhibit Altered Levels of Total and Active MMP-9 and Several Other Biomarkers in Serum and CSF Compared to Healthy Controls and Other Neurologic Diseases

**DOI:** 10.3390/ijms26188900

**Published:** 2025-09-12

**Authors:** Robert Bowser, Jiyan An, Kimberly Schwartz, Robert L. Sucholeiki, Irving Sucholeiki

**Affiliations:** 1 nVector, Inc, 124 W. Thomas Road STE 200, Phoenix, AZ 85013, USA; robert.bowser@nvector.com (R.B.); jiyan.an@nvector.com (J.A.); 2 Advansta, Inc., 2140 Bering Drive, San Jose, CA 95131, USA; kim@advansta.com; 3 Boston University School of Medicine, 72 E. Concord St., Boston, MA 02118, USA; rsuchol@bu.edu; 4 Aquilus Pharmaceuticals, Inc., 225 Mystic Valley Parkway, Winchester, MA 01890, USA

**Keywords:** matrix metalloproteinase, MMP-2, MMP-9, neurofilament light, creatinine, cystatin C, serum, Amyotrophic Lateral Sclerosis, cerebral spinal fluid, biomarker, AQU-118

## Abstract

Matrix metalloproteinases 2 and 9 (MMP-2, MMP-9) have been implicated in the pathogenesis of amyotrophic lateral sclerosis (ALS). However, their protein levels and correlation with other biomarkers are not well understood. We measured total and active MMP-2/-9 and additional biomarkers (creatinine, neurofilament light, cystatin C, and alkaline phosphatase) in the serum of people with ALS (ALS, n = 30) and compared their levels with age-matched healthy controls (HC, n = 20) and other neurological diseases (diabetic nephropathy, Alzheimer’s disease, Parkinson’s disease; n = 8 each). We also measured MMP-2/-9 in a set of CSF samples from ALS (n = 30) and age-matched other neurological diseases (OND, n = 14). Lastly, we measured the competitive binding behavior of a dual MMP-2/MMP-9 inhibitor, AQU-118, against active MMP-9 in situ within the serum of ALS. We found significantly elevated levels of both total MMP-9 protein (two studies, 7.5 and 9.5-fold; both *p* < 0.0001) and active MMP-9 (2.5-fold; *p* < 0.0001) in ALS serum compared to HC. Serum NfL was significantly elevated (6-fold, *p* < 0.0001) and serum creatinine was significantly decreased (40%, *p* < 0.0001) in ALS compared to HC. There were significantly decreased levels of MMP-2 (two studies, 26 and 33%; *p* < 0.001 and *p* = 0.0001, respectively) in the serum of ALS as compared to HC. ALS also had significantly higher active MMP-9 in serum than patients with Alzheimer’s disease and higher than Parkinson’s disease or diabetic nephropathy. We confirmed that active MMP-9 in ALS is fully available for proteolytic activity in both serum and CSF and can be inhibited using an MMP-2/-9 inhibitor. Active MMP-9 is systemically elevated in ALS and therefore a therapeutic target for ALS drug development.

## 1. Introduction

Matrix metalloproteinases (MMPs) are a large family of zinc-dependent endopeptidases [1]. As a family, MMPs can cleave virtually any component of the extracellular matrix to facilitate cell migration and affect cellular signaling, thereby regulating cell proliferation, differentiation, and cell death [2]. In addition to cleaving the structural components of the extracellular matrix, MMPs are involved in the processing and signaling of growth factors and their receptors, cytokines, chemokines, adhesion molecules, and a variety of other enzymes such as caspases [3,4]. A subset of MMPs, often referred to as gelatinases, are composed of MMP-2 and MMP-9 and are capable of collagen degradation. MMP-2 and MMP-9 protein levels have been reported to be altered in the blood, skin, and/or cerebrospinal fluid (CSF) of ALS patients or in animal models of ALS [5,6,7,8,9,10]. Beuche and colleagues, using enzyme-linked immunosorbent assay (ELISA), identified increased levels of MMP-9 protein in the serum but not the CSF of ALS when compared to controls [5]. A limited attempt was made using gelatin zymography to look at the active form of MMP-9, but only a few samples were examined, and no definitive conclusions were made. Lorenzl and colleagues measured the levels of MMP-2 and MMP-9 in the CSF, in addition to other biomarkers, and found no significant differences as compared to healthy controls [6]. Demestre and colleagues reported elevated levels of both the inactive (proform) and active forms of MMP-9 in the serum of ALS as compared to healthy controls [7]. Niebroj-Dobosz and colleagues reported significantly elevated levels of both MMP-9 and MMP-2 in the serum of ALS as compared to healthy controls. They also looked at the levels in CSF and found significantly elevated levels of MMP-2 but significantly decreased levels of MMP-9 [8]. Fang and colleagues found significantly elevated levels of MMP-9 in the skin and CSF of ALS, but not in serum, as compared to healthy controls [9]. They also examined MMP-2 and found no significant differences in serum, CSF, or skin as compared to healthy controls [9]. Overall, levels of MMP-9 were typically elevated in ALS serum but were reduced or showed no change in CSF, whereas MMP-2 levels exhibited more variability across studies.

The current study seeks to further define MMP-2 and MMP-9 protein levels and enzyme activity in the serum and CSF of ALS and compare their levels to those in the serum of age-matched, healthy controls (HCs) and CSF of other neurological diseases (OND). We also measured other biomarkers associated with ALS disease progression, including neurofilament light (NfL), serum creatinine, and serum cystatin C [11,12,13,14,15,16,17,18]. Most importantly, we sought to determine whether the active forms of MMP-2 and MMP-9 are present in serum and/or CSF of ALS and whether they are elevated compared to HC or OND. Answering this question is more challenging than it first may seem. Enzyme-linked immunosorbent assay (ELISA) cannot differentiate between the active and proforms of these metalloenzymes, so other methods such as gelatin zymography and/or Western blotting have been employed [5,6,7,8,9]. Those methods rely on the use of protein standards to confirm the presence or absence of these active forms via their band position on a gel that corresponds to their appropriate molecular weights. While these methods have allowed for the identification of the proforms of MMP-9 and MMP-2 with some degree of confidence, this has not been the case for their active forms. In many instances, protein standards for the active forms of MMP-2 and MMP-9 do not always align well with some of the observed bands presumed to be the active forms of MMP-2 and/or MMP-9 [19,20,21,22]. As such, these new observed bands have been attributed to being a partially cleaved isoform (i.e., formation of a 65 kD isoform of MMP-9 as a result of the cleavage of the C-terminal hemopexin portion), an active dimer/trimer form, or a charged variant. Other potential sources for these new bands have been attributed to being complexes composed of active MMP-9 and its corresponding tissue inhibitor of metalloproteinase-1 (TIMP1) or with neutrophil gelatinase-associated lipocalin (NGAL) [6,8,20]. TIMPs are proteins that act as natural inhibitors of MMPs and serve as regulators of their activity. It has therefore been assumed that an observed increase in TIMP1 and/or TIMP2 in a patient’s blood implies an inflammatory state in which the body is responding to an increase in the levels of the active forms of MMP-2 and/or MMP-9 [8]. NGAL is a small protein involved in the innate immune response, primarily functioning to bind and transport iron, and serves as an early biomarker for acute kidney injury and inflammation. An increase in the levels of NGAL has been thought by some to be a result of an increase in active MMP-9, resulting in the formation of an inactive NGAL/MMP-9 complex [23]. As such, rather than using methods employing gel electrophoresis, we utilized a method that uses a capture antibody for both the pro and active forms of MMP-2 and/or MMP-9, followed by the addition of a labeled peptide substrate that generates a fluorescent signal that is quantified upon cleavage by active MMP-2 or MMP-9. In this way, one can quantify the amount of active form without needing to know what configuration (i.e., isoform, dimer/trimer, or charged variant) the protein is in. If active MMP-2 and/or MMP-9 were present and found elevated in ALS, another goal of our work was to further elucidate the state of their active site(s) to determine if they are accessible or are blocked from further binding/activity. Lastly, if active forms of MMP-2 and/or MMP-9 were present in ALS, a final goal was to determine how their levels compare to those of other inflammatory and/or neurodegenerative diseases. While this study focuses on understanding the relationship between MMP-2/-9 and ALS, other neurodegenerative diseases, such as Parkinson’s and Alzheimer’s disease, have also been reported to have elevated levels of various MMPs [24]. As such, it was of interest to observe, in a limited manner, how any observed MMP changes in ALS compared to those in other neurodegenerative and non-neurological diseases, specifically diabetic nephropathy, which has an inflammatory component and may have bidirectional interactions between the kidneys and the nervous system.

## 2. Results

### 2.1. Total MMP-2 and MMP-9

**Figure 1:**
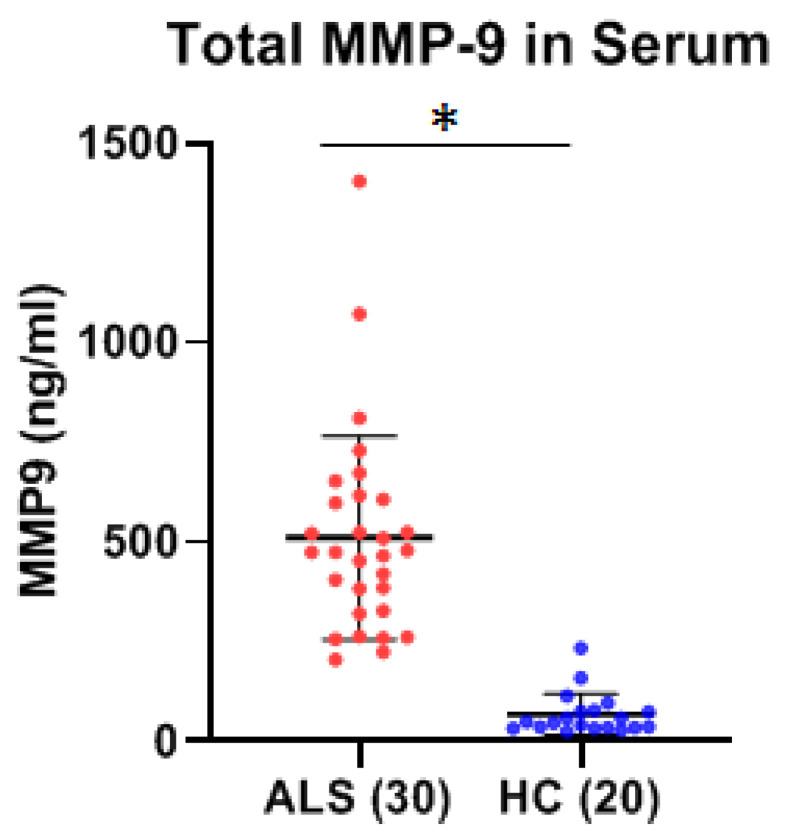
Comparison of serum MMP-9 levels between ALS (Group 1, Table 1) and healthy controls (HCs) (Group 2, Table 1). There was a significantly higher mean level of MMP-9 in the serum of ALS (510.2 ± 254.7 ng/mL) as compared to HC (67.5 ± 51.5 ng/mL), as determined by Welch’s *t*-test (* Significant, *p* < 0.0001, Cohen’s d = 2.21). There were no significant differences in the MMP-9 levels between males vs. females in either the control (*p* = 0.59) or ALS groups (*p* = 0.57).

**Figure 2:**
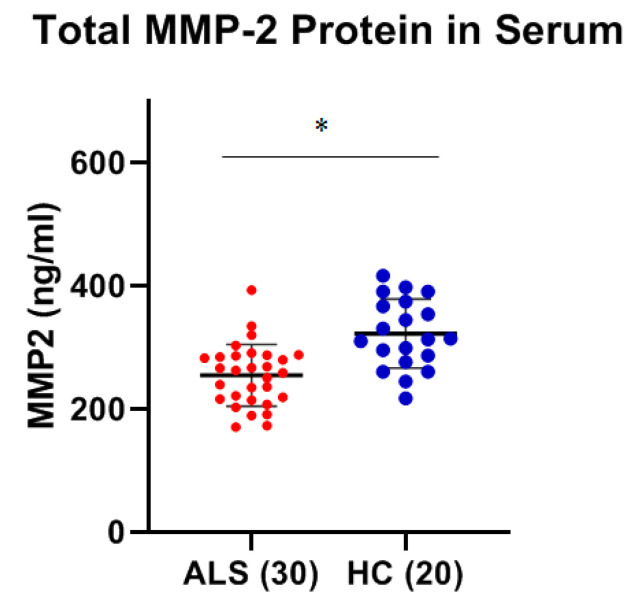
Comparison of serum MMP-2 levels between ALS (Group 1, Table 1) and healthy controls (HC) (Group 2, Table 1). Mean serum MMP-2 levels in ALS (254.6 ± 50.08 ng/mL) were significantly lower than HC (322.1 ± 56.02 ng/mL) as determined by Welch’s *t*-test (* Significant, *p* < 0.001, Cohen’s d = −1.28). There was no significant difference in MMP-2 levels between males vs. females in either the control (*p* = 0.1981) or ALS groups (*p* = 0.291).

Using ELISA, the levels of MMP-2 and MMP-9 were measured within the serum of thirty ALS (Group 1, Table 1: 18 males, 12 females; mean age ± SD, 59.4 ± 6.1 years) and twenty healthy, age-matched healthy controls (Group 2, Table 1, HC: 12 males, 8 females; 56.5 ± 2.3 years). There was a statistically significant increase in total mean MMP-9 concentration of greater than 7.5-fold within the serum of ALS as compared to that of HCs (*p* < 0.0001, Cohen’s d = 2.21) (Figure 1). There was a modest, but statistically significant, decrease (~26%) in the total mean MMP-2 concentration in the serum of ALS as compared to healthy controls (*p* < 0.001, Cohen’s d = −1.28) (Figure 2). No gender-specific differences were detected within the ALS or control groups for either MMP protein. The coefficient of variation (CV) for all samples was less than 15%, and typically less than 8%.

**Figure 3:**
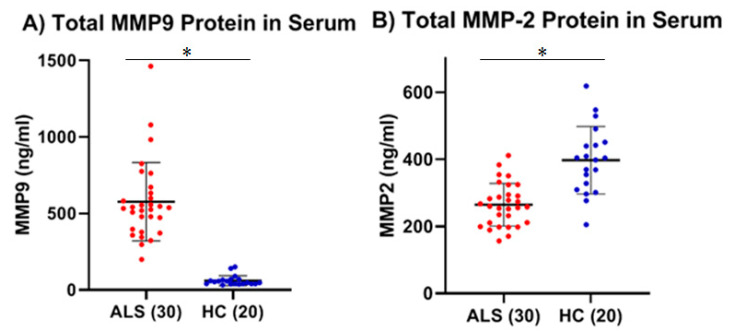
Comparison of total MMP-9 and MMP-2 protein levels between another group of ALS (Group 3, Table 1) and a group of healthy controls (HCs) (Group 5, Table 1). (**A**) Mean MMP-9 levels were 576.8 ± 255 ng/mL for ALS versus 60.6 ± 32.4 ng/mL for HC (* Significant, *p* < 0.0001, Welch’s *t*-test; Cohen’s d = 2.58). (**B**) Mean MMP-2 levels were 264.5 ± 63.6 ng/mL for ALS versus 397.3 ± 100.4 ng/mL for HC (* Significant, *p* = 0.0001, Welch’s *t*-test; Cohen’s d = −1.66).

**Figure 4:**
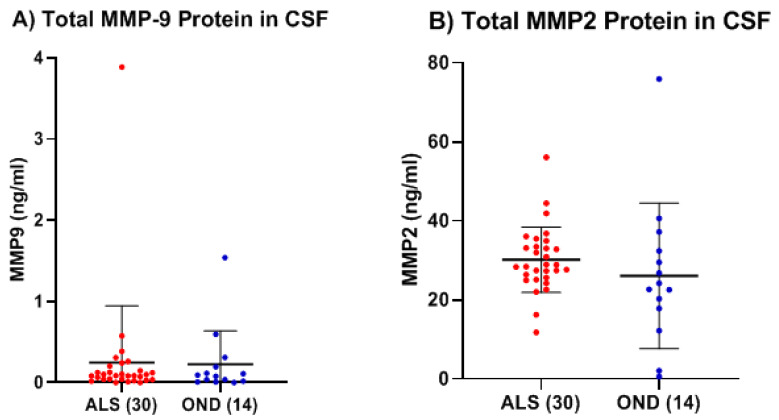
Comparison of total MMP-9 and MMP-2 protein levels in the CSF between another group of ALS (Group 4, Table 1) and a group of other neurological diseases (OND) (Group 6, Table 1). (**A**) Mean MMP-9 levels were 0.25 ± 0.70 ng/mL in ALS and 0.22 ± 0.41 ng/mL in OND (not significant, *p* = 0.95, Welch’s *t*-test; Cohen’s d = 0.04). (**B**) MMP-2 levels were 30.14 ± 8.30 ng/mL in ALS and 26.06 ± 18.44 ng/mL in OND (not significant, *p* = 0.44, Welch’s *t*-test; Cohen’s d = 0.33).

We then determined the levels of MMP-2 and MMP-9 in a separate validation set of thirty serum samples from ALS (Group 3, Table 1: 19 males, 11 females; mean age ± SD, 60.9 ± 7.9 years), with a matched set of CSF samples (Group 4, Table 1) from the same ALS, and compared their levels with a separate set of twenty serum samples from age-matched HC (Group 5, Table 1: 12 males, 8 females; 60.6 ± 5.5 years of age) and fourteen CSF samples from a group of age-matched individuals with other neurological diseases (Group 6, Table 1, OND: 8 males, 6 females; 63.6 ± 9.8 years of age). Consistent with our initial finding, we found a statistically significant increase in total mean MMP-9 concentration of greater than 9.5-fold within the serum of ALS compared to that of age-matched HCs (*p* < 0.0001, Cohen’s d = 2.58) (Figure 3A). Similarly, we replicated our initial result and found a statistically significant decrease (~33%) in the total mean MMP-2 concentration in the serum of ALS as compared to the new set of HC (*p* = 0.0001, Cohen’s d = −1.66) (Figure 3B). Within the CSF, there were very low levels of MMP-9 in both the ALS (0.25 ± 0.70 ng/mL) and OND groups (0.22 ± 0.41 ng/mL), which were not significantly different (*p* = 0.95, Cohen’s d = 0.04). (Figure 4A). While MMP-2 protein levels in the CSF of both ALS (30.14 ± 8.30 ng/mL) and OND (26.06 ± 18.44 ng/mL) were easily detected, there was no significant differences between the groups (*p* = 0.44, Cohen’s d = 0.33) (Figure 4B).

### 2.2. Neurofilament

**Figure 5:**
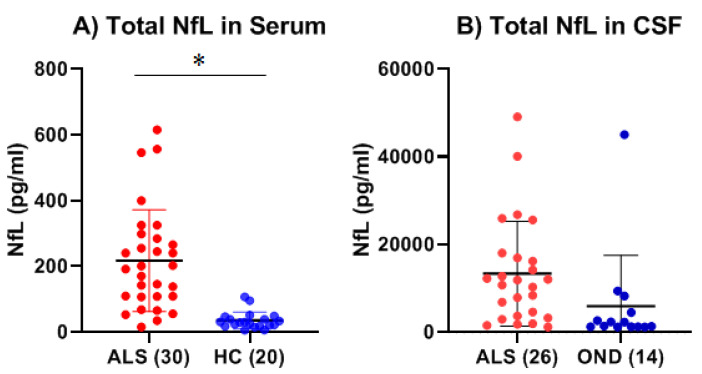
Neurofilament light (NfL) levels in the serum of ALS (Group 3, Table 1 and healthy controls (HC) (Group 5, Table 1) and in the CSF of ALS (Group 4, Table 1) and other neurological diseases (OND) (Group 6, Table 1). (**A**) There was a 6.1-fold increase in the mean levels of NfL (217.2 ± 154.6 pg/mL) in the serum of ALS as compared to HC (35.35 ± 25.93 pg/mL), which was statistically significant (* Significant, *p* < 0.0001, Welch’s *t*-test; Cohen’s d = 1.50). (**B**) There was a 2.2-fold increase in the mean levels of NfL (13,326 ± 11,962 pg/mL) in the CSF of ALS as compared to OND (n = 14) (5927 ± 11,572 pg/mL), which was not statistically significant (*p* = 0.067, Welch’s *t*-test; Cohen’s d = 0.63).

We next measured the levels of NfL in the serum of ALS and age-matched HC by ELISA. The levels of NfL were also measured in the CSF of ALS and compared to a set of age-matched OND. In serum, mean NfL levels were 6.1-fold higher in ALS (n = 30) (217.2 pg/mL ± 154.6 pg/mL) as compared to HC (n = 20) (35.35 pg/mL ± 25.93 pg/mL), which was statistically significant (*p* < 0.0001, Cohen’s d = 1.50) and expected based on prior studies (Figure 5A). In the CSF, mean NfL levels were 2.5-fold higher in ALS (n = 26) (13,326 ± 11,962 pg/mL) as compared to OND (n = 14) (5927 ± 11,572 pg/mL), but this difference was not statistically significant (*p* = 0.067, Cohen’s d = 0.63) (Figure 5B). We correlated the levels of NfL to either MMP-2 or MMP-9 in both the serum and CSF of ALS patients and detected no significant correlations between these two biomarkers.

### 2.3. Serum Creatinine

**Figure 6:**
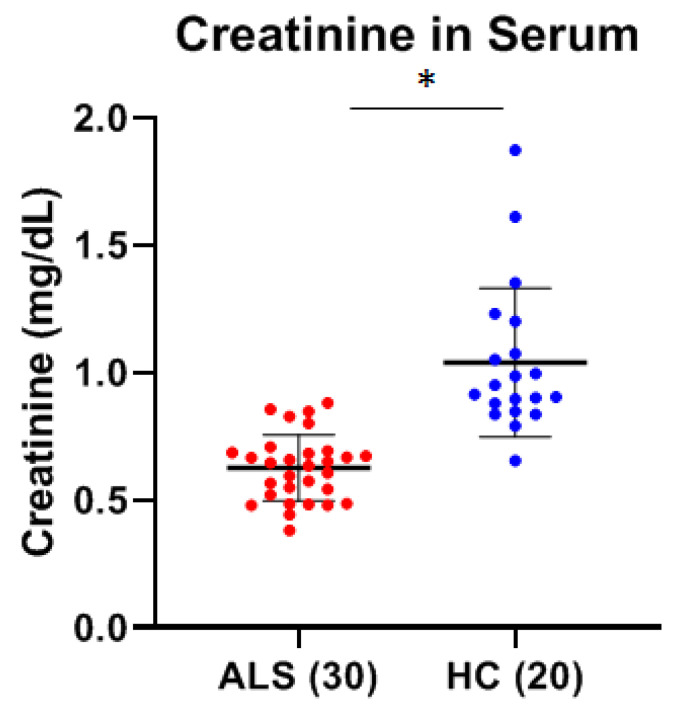
Comparison of serum creatinine in ALS (Group 3, Table 1) and healthy controls (HC) (Group 5, Table 1). There was a statistically significant decrease in the mean level of serum creatinine in ALS (0.6270 ± 0.1302 mg/dL) as compared to HC (1.0408 mg/dL ± 0.2924 mg/dL) (* Significant, *p* < 0.0001; Cohen’s d = −1.97).

We determined the creatinine concentration in the serum of both ALS and HC. There was a statistically significant (*p* < 0.0001, Cohen’s d = −1.97) decrease in the mean level of serum creatinine in ALS (0.6270 mg/dL ± 0.1302 mg/dL) as compared to HC (1.0408 mg/dL ± 0.2924 mg/dL) (Figure 6).

**Figure 7:**
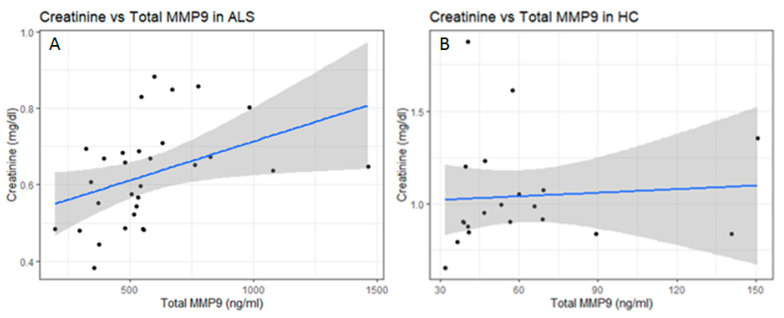
Correlation analysis between total serum creatinine and total serum MMP-9 in ALS (Group 3, Table 1) and healthy controls (HCs) (Group 5, Table 1). (**A**) There was a significant positive correlation between total serum MMP-9 and serum creatinine in ALS using both Pearson’s (*p* = 0.028) and Spearman’s rank (*p* = 0.005) analysis. (**B**) There was no significant correlation between total serum MMP-9 and serum creatinine in HC using Pearson’s (*p* = 0.766) and Spearman’s rank (*p* = 0.251) analysis.

We then assessed for any correlations between the levels of total MMP-2 or MMP-9 and serum creatinine. While there was no correlation between MMP-2 and creatinine levels, there was a statistically significant positive correlation between total serum MMP-9 and serum creatinine in ALS using both Pearson’s product–moment correlation (*p* = 0.028) and Spearman’s rank correlation (*p* = 0.005) analysis (Figure 7A). This positive correlation was not observed in healthy controls (Figure 7B). There was also no correlation between the levels of serum NfL and creatinine in ALS.

### 2.4. Serum Cystatin C and Estimated Glomerular Filtration Rate

**Figure 8:**
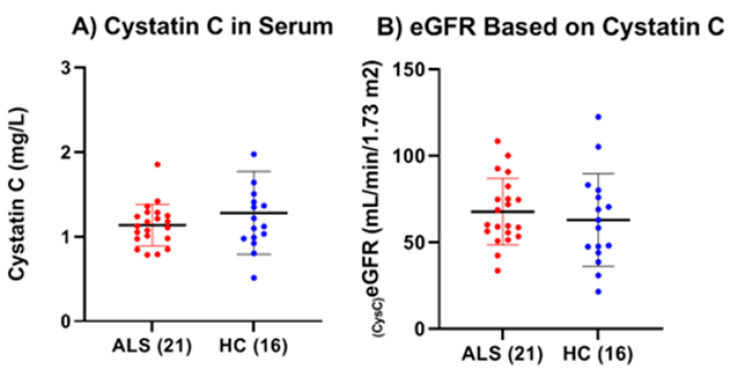
Comparison of total serum cystatin C and calculated eGFR in ALS (Group 3, Table 1) and healthy controls (HCs) (Group 5, Table 1). (**A**) There was no significant difference in the mean serum cystatin C levels between ALS (1.14 ± 0.25 mg/L) and HC (1.28 ± 0.49 mg/L) (*p* = 0.2497, Cohen’s d = −0.39). (**B**) There were no significant differences in cystatin C eGFR between ALS (67.70 ± 19.20 mL/min/1.73 m^2^) and HC (62.94 ± 26.72 mL/min/1.73 m^2^) (*p* = 0.5317, Cohen’s d = 0.21).

We determined the cystatin C concentration in the serum of both ALS (n = 21) and HC (n = 16). There was no statistically significant difference between the mean cystatin C levels in ALS (1.14 ± 0.25 mg/L) and HC (1.28 ± 0.49 mg/L) (*p* = 0.25, Cohen’s d = −0.39) (Figure 8A). Based on the experimentally determined serum cystatin C levels, we then calculated an estimated glomerular filtration rate (eGFR) for each of the ALS and HC [25]. There were also no significant differences in eGFR between ALS and HC (*p* = 0.532, Cohen’s d = 0.21) (Figure 8B).

**Figure 9:**
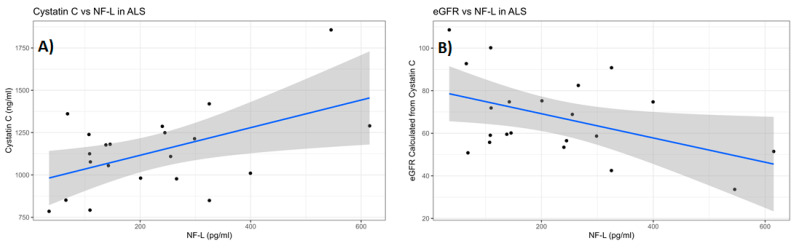
Analysis of serum NfL and serum cystatin C levels and the corresponding eGFR in ALS (Group 3, Table 1). (**A**) There was a significant positive correlation between serum NfL and serum cystatin C (Pearson’s *p* = 0.018, Spearman’s rank *p* = 0.1423). (**B**) There was a significant negative correlation between serum NfL and the cystatin C eGFR in ALS (Pearson’s *p* = 0.037, Spearman’s rank *p* = 0.132).

We found no correlation between total MMP-2 or MMP-9 and cystatin C or eGFR levels. However, we found a significant positive correlation between serum NfL levels and cystatin C among ALS (Pearson’s *p* = 0.018, Spearman’s rank *p* = 0.143) and among HC (Pearson’s *p* = 0.004, Spearman’s rank *p* = 0.491) and a significant negative correlation of the corresponding eGFR (Pearson’s *p* = 0.037, Spearman’s rank *p* = 0.132) in ALS, but not in HC (Pearson’s *p* = 0.098, Spearman’s rank *p* = 0.534). (Figure 9A,B).

### 2.5. Active MMP-2 and MMP-9 in Serum

**Figure 10:**
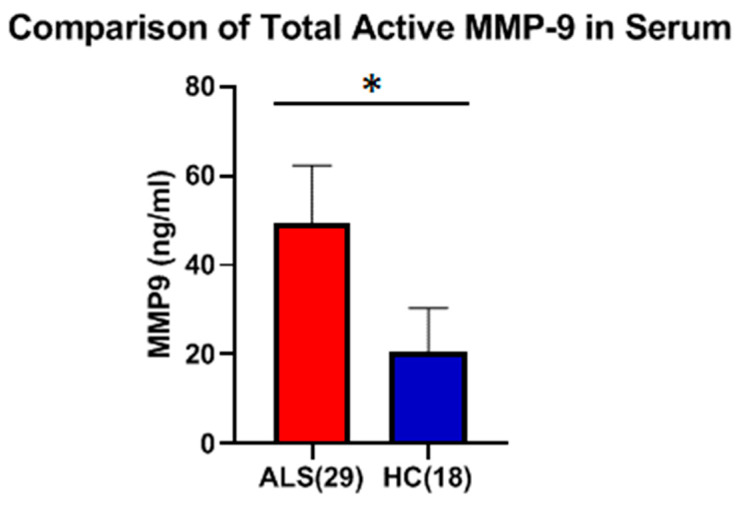
Comparison of total active MMP-9 protein in serum between ALS (Group 3, Table 1) and healthy controls (HCs) (Group 5, Table 1). Active MMP-9 levels in serum were 50.6 ± 12.4 ng/ml for ALS versus 20.97 ± 11.29 ng/ml for HC (* Significant, *p* < 0.0001, Welch’s *t*-test; Cohen’s d = 2.47).

The level of active MMP-9 in serum was measured using the Fluorokine E assay, and the mean (50.6 ng/mL ± 12.4 ng/mL) level in ALS serum was found to be significantly higher (*p* < 0.0001, Cohen’s d = 2.47) as compared to HC (20.97 ng/mL ± 11.29 ng/mL), with a mean increase of 2.4-fold (Figure 10).

**Figure 11:**
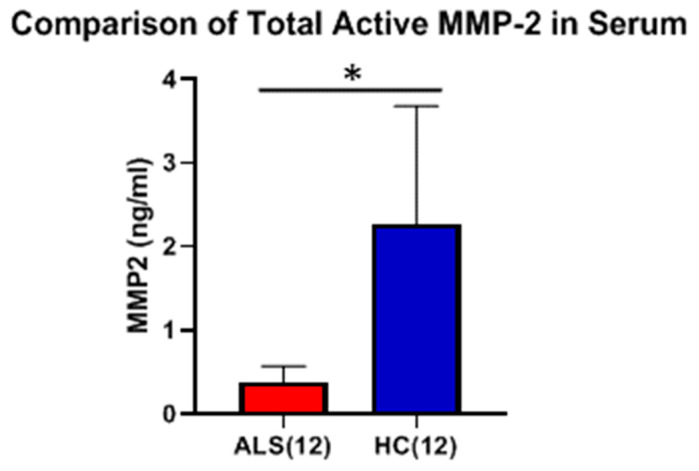
Comparison of total active MMP-2 protein in serum between ALS (Group 3, Table 1) and healthy controls (HCs) (Group 5, Table 1). Mean active MMP-2 levels in serum were 0.4 ± 0.2 ng/ml for ALS versus 2.3 ± 1.4 ng/ml for HC (* Significant, *p* < 0.0001, Welch’s *t*-test; Cohen’s d = −3.05).

The level of active MMP-2 was measured using the Quickzyme assay, which requires the active form of MMP-2 to cleave and activate a separate Pro-Enzyme, which then cleaves a FRET (fluorescence resonance energy transfer) peptide. Using this assay system, the levels of active MMP-2 in ALS serum were found to be significantly lower than HC serum (Figure 11). However, the levels of active MMP-2 for both ALS and HC were found to be quite low, at the lower limits of detection for the assay.

**Figure 12:**
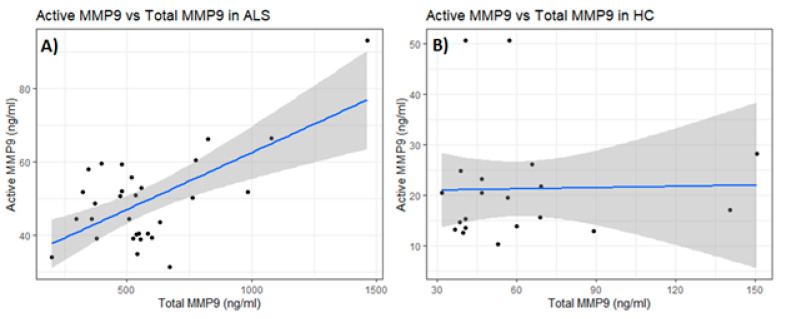
Correlation analysis between total and active MMP-9 for both ALS (Group 3, Table 1) and healthy controls (HCs) (Group 5, Table 1). (**A**) There was a significant, positive correlation between the levels of total and active MMP-9 in the serum of ALS (Pearson’s correlation, *p* = 0.0002, Spearman’s rank *p* = 0.267). (**B**) There was no significant correlation between the levels of total and active MMP-9 in the serum of HC (Pearson’s *p* = 0.919, Spearman’s rank *p* = 0.447).

There was a significant positive correlation (Pearson’s correlation, *p* = 0.0002, Spearman’s rank *p* = 0.27) between total and active MMP-9 in the serum of ALS (Figure 12A). However, this correlation was not observed among the serum of HC (Pearson’s *p* = 0.9192, Spearman’s rank *p* = 0.45) (Figure 12B), nor was it observed for total and active MMP-2.

### 2.6. Active MMP-2 and MMP-9 in CSF

**Figure 13:**
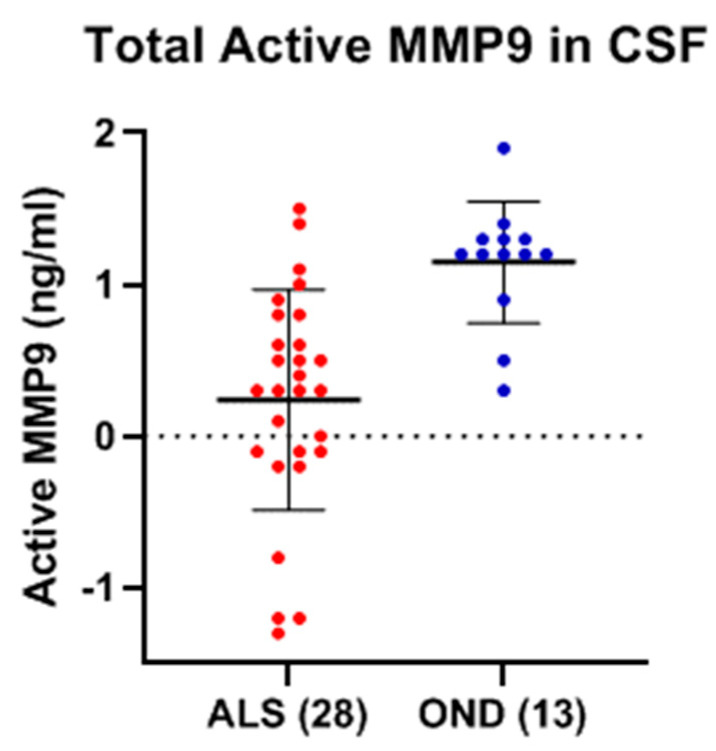
Mean level of active MMP-9 in the CSF of ALS (Group 4, Table 1) (0.23 ± 0.74 ng/ml) and OND (Group 6, Table 1) (1.15 ± 0.40 ng/ml). The levels measured were at the lower detection limit of the assay.

Low levels of active MMP-9 were detected in the CSF of both ALS and OND (Figure 13). While there appeared to be a statistically significant decrease in the mean level of active MMP-9 in ALS (0.23 ± 0.74 ng/ml) as compared to OND (1.15 ± 0.40 ng/ml) (*p* < 0.0001; Cohen’s d = −1.41), their levels were at the lower level of detection of the assay and deemed too low for the statistical analysis to have any degree of confidence (Figure 13). There were also very low levels of active MMP-2 detected in the CSF of both ALS and OND, but their levels were too low (<1 ng/ml) for any statistical comparisons to be made.

### 2.7. Active MMP-9 Inhibition

**Figure 14:**
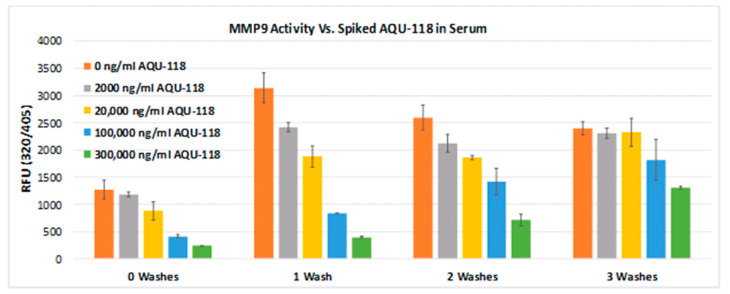
MMP-9 protease activity in serum of ALS (Specific sample from Group 7, Table 1) with and without spiked AQU-118 at different inhibitor concentrations and after 0–3 washes (performed in duplicate). Assay was performed using the Fluorokine E MMP-9 Assay Kit (performed in duplicate).

We next evaluated whether the active form of MMP-9 in a human biofluid has a freely accessible active site for protease activity or is permanently blocked by some endogenous high-affinity binder. This was examined by both exposing the active form in situ with a cleavable fluorescent peptide and with a small molecule semi-selective inhibitor of both MMP-2 and MMP-9 (MMP-2 full-length IC50 = 3.8 nM, MMP-9 full-length IC50 = 14.6 nM) with the designation AQU-118 [26]. By exposing a serum sample to varying concentrations of AQU-118, one can determine whether the inhibitor can compete at the active site, thereby modulating the peptidase activity as measured using the Fluorokine E assay. Using the Fluorokine E assay, the level of MMP-9 activity was measured within a serum sample from ALS, performed in parallel with a series of identical serum samples spiked with varying concentrations of the inhibitor AQU-118, to see if one could observe any differences in protease activity between those with and without the spiked inhibitor. We also compared the number of washes (from zero to three) that were applied as part of the assay protocol to see how this would affect the level of activity. First, we looked at just exposing bound active MMP-9 to a fluorescently labeled peptide substrate and found proteolytic activity (Figure 14). Next, we found that spiked concentrations of AQU-118 exhibited significantly lower activity as compared to the non-spiked samples, even after applying a number of wash steps (Figure 14). Inhibition of the bound active MMP-9 followed a concentration-dependent manner, with increasing concentrations of spiked AQU-118 eliciting an increase in MMP-9 inhibition. When no wash step was performed, the level of activity of the non-spiked serum sample was observed to be less than when there was one wash step. This may be due to concentration effects impacting the fluorescence measurement or may indicate that at the initial equilibrium state, a portion of the MMP-9 active sites are blocked, and that further washes prevent any MMP-9-bound complex from forming a new equilibrium state that frees up additional active sites. Lastly, the fact that AQU-118 was able to not only inhibit MMP-9 when there were no wash steps, but that its inhibitory effects persisted after as many as two subsequent washes indicates that, if there are biomolecules that are binding to the active site in situ to active MMP-9, their binding affinities or concentrations are lower than that of the binding affinity or concentrations of AQU-118. However, it is clear that some portion of the active form of MMP-9 is freely available to engage in proteolytic activity, and that exposing its active site to an external inhibitor can block this activity.

### 2.8. Comparison of Biomarkers Between ALS and Other Diseases

**Figure 15:**
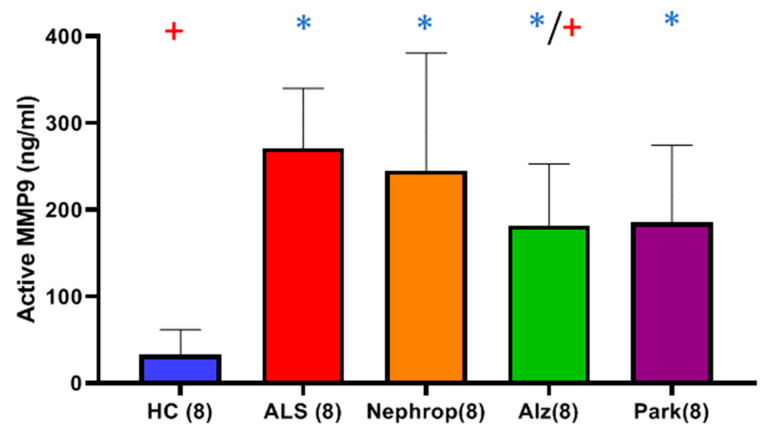
Comparison of mean active MMP-9 in a set (n = 8 each) of serum samples from ALS (Group 7) (271.25 ± 68.86 ng/ml), diabetic nephropathy (Nephrop) (Group 9) (245.63 ± 135.10 ng/ml), Alzheimer’s disease (Alz) (Group 10) (181.88 ± 71.11 ng/ml), Parkinson’s disease (Park) (Group 11) (185.63 ± 88.94 ng/ml), and healthy controls (HC) (Group 8) (33.13 ± 28.65 ng/ml). Analysis (*t*-test with Benjamini–Hochberg correction) indicates the ALS (* Significant, *p* < 0.0001), Nephrop (* Significant, *p* = 0.007), Alz (* Significant, *p* = 0.002), and Park (* Significant, *p* = 0.0050) groups all exhibited significantly higher mean active MMP-9 as compared to HC (*). ALS (+) had the highest mean level of active MMP-9 out of all of the groups, which was significantly higher than that in Alz (+ Significant, *p* = 0.0459), and HC (+ Significant, *p* < 0.0001) and higher than Nephrop (*p* = 0.714) and Park (*p* = 0.084).

While the levels of total and active MMP-9 were significantly higher in the serum of ALS as compared to HC, we explored if elevated levels were unique to ALS or could also be observed in other diseases that have a known inflammatory component. We measured levels of active MMP-9 within a small set (n = 8) of serum samples taken from people diagnosed with diabetic nephropathy (four males, four females, 61 ± 7 years of age), Alzheimer’s disease (four males, four females, 60 ± 5 years of age) and Parkinson’s disease (four males, four females, 62 ± 3 years of age), and compared them to a subset (n = 8) of closely age-matched serum samples taken from the second group of ALS (five males, three females, 65 ± 8 years of age) and HC (four males, four females, 61 ± 6 years of age) samples. The results showed that all of the disease groups exhibited significantly higher mean active MMP-9 as compared to HC (Figure 15). The ALS group exhibited the highest mean levels of active MMP-9 of all of the groups, which was significantly higher than that of Alzheimer’s (*p* = 0.023), and HC groups (*p* < 0.0001), and higher than the nephropathy (*p* = 0.714.) or Park (*p* = 0.084) groups (Figure 15).

**Figure 16:**
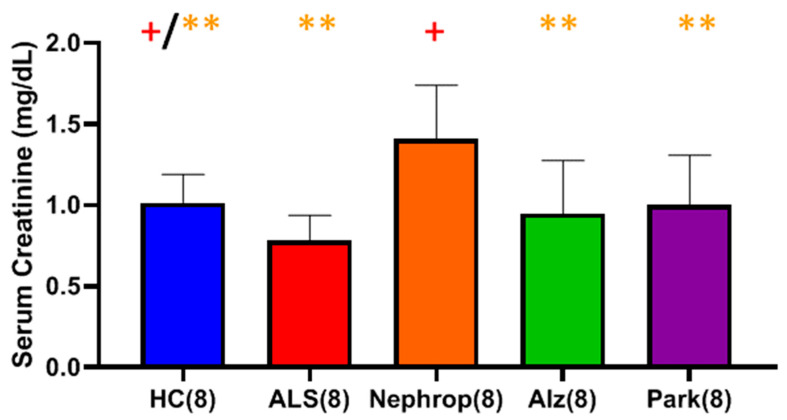
Comparison of serum creatinine in a set (n = 8 each) of serum samples from ALS (Group 7), diabetic nephropathy (Nephrop) (Group 9), Alzheimer’s disease (Alz) (Group 10), Parkinson’s disease (Park) (Group 11), and healthy controls (HC) (Group 8). Analysis (*t*-test with Benjamini–Hochberg correction) indicates that the ALS (+) group had the lowest mean level of serum creatinine compared to all other groups (0.78 ± 0.16 mg/dL), which was statistically significant with respect to the HC (1.02 ± 0.17 mg/dL) (+ Significant, *p* = 0.0345) and Nephrop (1.41 ± 0.33 mg/dL) (+ Significant, *p* = 0.0064) groups, and not statistically significant with respect to the Alz (0.95 ± 0.33 mg/dL) (*p* = 0.3094) and Park (1.00 ± 0.31 mg/dL) (*p* = 0.1691) groups. The Nephrop group (**) had the highest mean level of serum creatinine of all groups, which was significantly higher compared to the HC (** Significant, *p* = 0.0345), ALS (** Significant, *p* = 0.0064), Alz (** Significant, *p* = 0.0345), and Park (** Significant, *p* = 0.0447) groups.

We measured serum creatinine in these same samples and found that the ALS group had the lowest mean level of serum creatinine of all groups, which was significantly lower than the HC and diabetic nephropathy groups (Figure 16). Conversely, the diabetic nephropathy group had the highest mean level of serum creatinine, which was significantly higher than all of the other groups (Figure 16).

**Figure 17:**
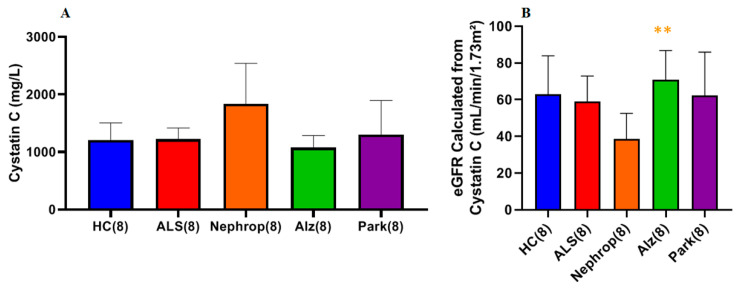
Comparison of serum cystatin C and calculated eGFR in a set (n = 8 each) of serum samples from ALS (Group 7), diabetic nephropathy (Nephrop) (Group 9), Alzheimer’s disease (Alz) (Group 10), Parkinson’s disease (Park) (Group 11), and healthy controls (HC) (Group 8). (**A**) Analysis (*t*-test with Benjamini–Hochberg correction) indicates the ALS group had a mean level of cystatin C (1.22 ± 0.19 mg/L) that was not significantly different from any of the other disease groups. The Nephrop group had the highest mean levels of cystatin C of all groups (1.84 ± 0.70 mg/L), which was higher but not statistically significant than the HC (1.21 ± 0.31 mg/L) (*p* = 0.15), ALS (1.22 ± 0.19 mg/L) (*p* = 0.15), Alz (1.08 ± 0.21 mg/L) (*p* = 0.15) and Park (1.30 ± 0.59 mg/L) (*p* = 0.31) groups. (**B**) Analysis (*t*-test with Benjamini–Hochberg correction) of the calculated eGFR for each group showing that Nephrop group had the lowest mean eGFR (38.47 ± 14.11 mL/min/1.73 m^2^) of all of the groups, which was significantly lower than Alz (70.90 ± 15.85 mL/min/1.73 m^2^) (** Significant, *p* = 0.00721), trending significance to ALS (58.97 ± 13.87 mL/min/1.73 m^2^) (*p* = 0.0549), HC (62.88 ± 21.11 mL/min/1.73 m^2^) (*p* = 0.0613), and Park (62.26 ± 23.69 mL/min/1.73 m^2^) (*p* = 0.0802) groups.

**Figure 18:**
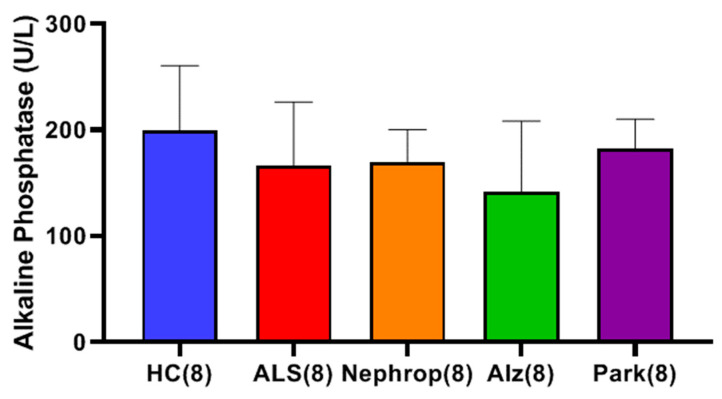
Comparison of mean serum alkaline phosphatase (ALP) in a set (n = 8 each) of serum samples from ALS (Group 7), diabetic nephropathy (Nephrop) (Group 9), Alzheimer’s disease (Alz) (Group 10), Parkinson’s disease (Park) (Group 11), and healthy controls (HCs) (Group 8). Analysis (*t*-test with Benjamini–Hochberg correction) indicates the HC group had the highest mean ALP (199.38 ± 61.24 U/L) of all of the groups, but was not significant with respect to the ALS (166.63 ± 59.69 U/L) (*p* = 0.560), Nephrop (169.50 ± 30.84 U/L) (*p* = 0.560), Alz (141.88 ± 66.55 U/L) (*p* = 0.560), and Park (182.63 ± 27.51 U/L) (*p* = 0.560) groups.

We next measured the serum cystatin C level in the same sample set and calculated their corresponding eGFR. We found that the nephropathy group exhibited the highest mean serum cystatin C level, which was higher (though not statistically significant) than that of the HC (*p* = 0.15), ALS (*p* = 0.15), Alzheimer’s (*p* = 0.15) and Parkinson’s (*p* = 0.31) groups (Figure 17A). Examining eGFR, the cystatin C eGFR of the ALS group was not significantly different from that of healthy controls (Figure 17B). The nephropathy group exhibited the lowest mean eGFR level of all of the groups, which was significantly lower compared to the Alzheimer’s (*p* = 0.007) group and lower (though not statistically significant) than that of the HC (*p* = 0.061), ALS (*p* = 0.055), and Parkinson’s (*p* = 0.080) groups (Figure 17B). Additionally, there were no observed correlations between the levels of serum cystatin C and the levels of total and/or active MMP-9 in ALS or HC. Lastly, we measured levels of serum alkaline phosphatase (ALP) (Figure 18). Examination of each of the groups found that HC had the highest mean ALP of all of the groups but was not significantly different with respect to the ALS (*p* = 0.560), Nephrop (*p* = 0.560), Alz (*p* = 0.560), and Park (*p* = 0.560) groups.

## 3. Discussion

MMP-2 and MMP-9 exist in human biofluids and tissues as a mixture of both active and inactive forms (i.e., proforms). A cysteine located in the N-terminal pro-domain of the proform binds to the zinc atom in the active site of the enzyme, thus maintaining latency. Activation of the proform requires a disruption of the cysteine linkage with the zinc atom (via peptide fragment cleavage containing the cysteine or oxidation of the cysteine thiol), thus exposing the catalytic site. While it is rather straightforward to measure both the active forms and proforms via ELISA, differentiating the levels of the active form from that of the proform is somewhat challenging. Work by Aquilus and others have found that techniques such as Western blotting and gelatin zymography cannot, in many cases, adequately determine the levels of the various active forms of MMP-2 and MMP-9, as their locations on either Western or zymography gels do not always align with commercial standards [27]. However, a different method was used for this study that utilizes a combination of selective antibody capture of both the pro and active forms of MMP-2 or MMP-9, followed by measuring the fluorescence of a peptide substrate cleaved by the bound active forms to thereby quantify their levels. In this way, one does not need to know the exact make-up of the active form (i.e., dimer or monomer with partial or total removal of C-terminal hemopexin-like domain) but only that it is functionally capable of proteolytically cleaving the FRET-labeled peptide.

For example, to measure active MMP-9 from a potential mixture containing various active forms of MMP-9 mixed with ProMMP-9 within serum or CSF, a Fluorokine E assay was used, which first exposes an immobilized anti-MMP-9 antibody that recognizes and captures all the active variants of MMP-9 and ProMMP-9 forms. Once all the forms have been captured, a FRET (fluorescence resonance energy transfer) peptide that is labeled with a fluorescence recorder is exposed to the bound enzymes. Only the active forms can cleave the FRET peptide, which in turn generates fluorescence upon peptide cleavage. The fluorescence measurement is then related to the concentration of the active forms of MMP-9 in the sample. By using a combination of standard ELISA for measuring total MMP-9 and the Fluorokine E assay for measuring only active MMP-9, one can obtain a clear picture of the levels of active and non-active forms. In this same manner, the level of active MMP-2 was measured using a slightly different assay (Quickzyme assay), which, like the Fluorokine E assay, uses an antibody to capture all of the active and non-active forms of MMP-2. However, unlike the Fluorokine E assay, the Quickzyme assay requires the active forms of MMP-2 to cleave and activate another proprietary Pro-Enzyme, which then cleaves the FRET peptide. This extra step enhances the specificity of the assay but also reduces the assay’s sensitivity.

Using these approaches, the levels of total and active MMP-9 were measured in both the serum and CSF in ALS (Group 1, Table 1) and compared to HC (Group 2, Table 1). The results showed that there was a significant increase in both total (two studies, 7.5- and 9.5-fold; both *p* < 0.0001) and active (2.4-fold; *p* < 0.0001) MMP-9 in the serum of ALS as compared to HC. Increased serum MMP-9 in ALS was confirmed in a separate cohort of serum samples (Figure 3). There was a positive correlation via Pearson’s correlation (Pearson’s *p* = 0.0002, Spearman’s rank *p* = 0.27) between the levels of total and active MMP-9 in ALS (Group 3, Table 1) that was not observed in HC (Group 5, Table 1). In the CSF, low levels of both total and active MMP-9 were measured in both ALS (Group 4, Table 1) and OND (Group 6, Table 1), but there were no significant differences between the two groups, nor any correlation between their respective active and total MMP-9 levels, either in the ALS or OND groups. Comparing CSF from a specific neurological disease such as ALS to an OND group representing various other neurological diseases can increase biomarker variability due to diverse underlying pathologies. This added variability may obscure disease-specific signals, reducing the ability to detect meaningful differences. However, comparing MMP-9 in ALS with an OND group helps assess the specificity of a biomarker by determining whether it can distinguish the target disease, in this case, ALS, from other neurological conditions with overlapping symptoms or if the biomarker reflects a broader neurogenerative or inflammatory processes. From our results it is clear that the levels of total MMP-9 are not specific to ALS and that other neurological diseases are undergoing processes that are generating similar MMP-9 levels. How do these levels compare with the CSF of healthy adults? CSF from age-matched healthy controls are rare and were not available for this study but could be explored in future studies. However, in prior reports in the literature, where ELISA techniques had been optimized for the detection of MMP-9 in the CSF among recruited confirmed healthy adults (n = 27, median age = 33 years, range = 26–43 years of age), the levels of MMP-9 ranged from 0.156 to 0.189 ng/ml, which is lower than our observed mean MMP-9 levels for either the ALS (0.25 ng/ml) or OND (0.22 ng/ml) groups [28].

One observes a small, but significant, decrease in total MMP-2 (two studies, 26% and 33%; *p* < 0.001 and *p* = 0.0001, respectively) in the serum of ALS versus HC using standard ELISA. While active MMP-2 was detected in the serum of ALS using the Quickzyme assay, the levels were very low and not statistically different from HC. Unlike MMP-9, there was no observed correlation in serum between the levels of total and active MMP-2 in ALS nor in HC. In the CSF, the levels of total MMP-2 in ALS were not statistically different from those of OND. The levels of active MMP-2 in the CSF of both ALS and HC, while detectable in some cases, were too low to be measured with any degree of confidence. It is possible that the low levels of active MMP-2 measured in both serum and CSF may be due to the unique nature of the assay used, as it relies on an additional enzymatic step (compared to the assay used to measure active MMP-9), which reduces the overall sensitivity of the measurement.

The finding that one can detect active MMP-9 in both the serum and CSF of ALS, with a significantly elevated level of active MMP-9 in the serum compared to HC, provides a potential pathway by which critical proteins may be broken down in the ALS disease state. However, as was mentioned in the introduction, there has been speculation that any active MMP-9 would most likely be regulated via the binding of an inhibitor protein such as TIMP1 and/or NGAL. Because the standard assay conditions for the Fluorokine E assay utilize several wash steps that result in the release of any potential non-covalent binders from the active site, it is possible that regulator proteins could be bound to the MMP-9 active site but are simply washed off. As such, we sought to determine whether there is any indication of the active site being blocked by exposing the active MMP-9 protein without any wash steps as well as to a competitive inhibitor in situ. By varying the number of washes and concentration of spiked inhibitor and comparing it to the resulting fluorescence from the cleaved peptide, one can discern information about the in situ state of the MMP-9 active site. Focusing on our data where there are no washes and no spiked inhibitor, one observes some level of fluorescence as a result of peptide cleavage. This clearly implies that there is some percentage of MMP-9 active sites that are not blocked in any significant manner and are capable of protease activity. After one wash and no spiked inhibitor, one observes a significant rise in fluorescence, which at first glance may indicate that potential binders are equilibrating out of the active site. However, such a conclusion must be tempered by the fact that concentration effects could also be impacting the level of fluorescence. In other words, the washes may be removing biological components that may be quenching the fluorescence generated by the peptide cleavage step. One also observes that after the first wash, some fluorescence is lost, indicating that possibly some of the antibody-bound active MMP-9 may be dislodging and washing away. Our AQU-118 data suggests that active MMP-9 is capable of being inhibited in situ in a concentration-dependent manner by the dual active MMP-2/9 inhibitor, AQU-118. This result clearly indicates that the active site is not strongly blocked by the binding of a high-affinity ligand and is both capable of proteolytic activity and amenable to inhibition, making a case for its regulation via dosing with an MMP inhibitor such as AQU-118.

We detected significantly elevated serum NfL levels in ALS patients compared to healthy controls (HC) by as much as 6-fold. This is consistent with the existing literature identifying NfL as a sensitive and reliable biomarker for monitoring ALS progression [12]. Its clinical significance was further underscored when it became the first biomarker used by the FDA to support the approval of Tofersen, a treatment for SOD1-ALS [13]. However, no correlation was observed between the levels of total or active MMP-9 and the levels of NfL in either serum or CSF. One possible explanation is that these biomarkers originate from distinct inflammatory processes occurring in ALS. Although this may seem surprising, it aligns with findings from other studies on neuroinflammation. For example, in cases of optic neuritis associated with multiple sclerosis, elevated levels of both total MMP-9 and NfL were observed in the CSF of patients compared to healthy controls [28]. However, while a strong correlation was found between MMP-9 and other markers such as CXCL10, no correlation was observed between MMP-9 and NfL. This led the authors to speculate that separate but parallel inflammatory processes were occurring [28]. In the CSF, we did not observe a significant difference in NfL levels between ALS and OND groups, but these levels are significantly higher than those previously reported for NfL in the CSF of HC. For example, in one study, NfL in the CSF among recruited confirmed healthy adults (n = 27, median age = 33 years, range = 26–43 years of age), ranged from 1000 to 8532 pg/ml, which is lower than our observed mean NfL levels for the ALS (13,326 pg/ml) group [28]. In another study where the recruited confirmed healthy adults (n = 75) were closer of age (mean age = 68.3 years ± 9.4 years) to the age in our study (mean 60.6 ± 5.5 years), the levels of NfL in the healthy control group ranged from 398 to 777 pg, which is even lower than the mean NfL observed in our study [29].

Regarding the origins of the elevated levels of total and active MMP-9 in the serum, there has been speculation that a possible source of MMP-9 may be derived from the CNS through the breakdown of the blood–brain barrier [30]. However, this is unlikely to be a driving factor since the quantity of total MMP-9 observed in the CSF of ALS is still only 0.04% of that found in serum, which seems rather low to account for such a large quantity in the serum. The markedly higher level of total MMP-9 in serum suggests that the reverse—MMP-9 migrating from serum to CSF—could be taking place. This is unlike the quantity of NfL, which was observed to be just over 60-fold higher in the CSF than in serum. Barring the possibility that the elevated levels of total and active MMP-9 in serum are solely derived from the CNS, it is more probable that the observed elevated levels are derived from peripheral tissues such as degenerating muscle. Others have speculated on muscle as a significant source of MMP-9, but evidence has been lacking [5,6]. It has been known that matrix metalloproteinases, such as MMP-2 and MMP-9, play a critical role in the homeostasis and maintenance of myofiber functional integrity and that inhibition of MMP-9 improves myofiber regeneration in rodent models of Duchenne muscular dystrophy [31,32,33]. As such, we aimed to determine the level of muscle degeneration in ALS and see if this degeneration is related to the levels of total and/or active serum MMP-9.

One way to determine muscle degeneration is to follow its mass via creatinine levels in the serum. Serum creatinine is a byproduct of the nonenzymatic breakdown of creatine phosphate, an energy source found in muscle tissue. It is produced at a relatively constant rate, with approximately 1–2% of muscle creatine converted to creatinine each day. Once formed, creatinine is transported through the bloodstream to the kidneys for excretion. Its levels in the blood are influenced primarily by an individual’s muscle mass and renal status [14]. Several studies have reported significantly lower serum creatinine levels in ALS as compared to HC, with some studies noting reductions as early as one year before ALS diagnosis [14,15,16]. These findings suggest that serum creatinine is a sensitive marker of ALS disease progression, with lower levels reflecting the loss of muscle mass. Our results confirmed that there is a statistically significant decrease of around 40% in the mean level of serum creatinine in ALS as compared to HC (*p* < 0.0001). We observed no significant correlations between serum creatinine levels and NfL in ALS or in HC. We did observe a significant positive correlation between total serum MMP-9 levels and serum creatinine levels in ALS (Pearson’s *p* = 0.028, Spearman’s rank *p* = 0.005) that was not observed among HC (Pearson’s *p* = 0.77, Spearman’s rank *p* = 0.25). However, no correlation (positive or negative) was observed for active MMP-9 and serum creatinine. As such, a mechanism that can explain the positive correlation between total MMP-9 and serum creatinine within muscle remains unclear at present, though several possibilities exist. For instance, in the context of skeletal muscle injury, elevated serum MMP-9 levels may result from degranulating neutrophils that infiltrate the necrotic muscle early on, followed by macrophages that contribute additional MMP-9 as the inflammatory response progresses [34]. This increase in MMP-9 reflects the breakdown of the local extracellular matrix. At the same time, muscle cell damage leads to a rise in serum creatinine, as it is continuously produced from muscle creatine and released when muscle fibers are lysed [35]. In such cases, the positive correlation between MMP-9 and serum creatinine is likely a consequence of both muscle breakdown and inflammatory infiltration.

Another potential source for the elevated levels of total and active MMP-9 is the kidney via renal dysfunction. It is known that in ALS there is a slightly higher prevalence (~10%) of chronic kidney disease (CKD) [36], whereby persistent inflammation and oxidative stress stimulate the upregulation of MMP-9 expression by immune cells such as macrophages, as well as renal tubular epithelial cells [37]. This overexpression contributes to ongoing renal tissue remodeling and fibrosis, worsening kidney function. Simultaneously, as kidney function declines, the glomerular filtration rate (GFR) decreases, leading to the accumulation of creatinine in the blood [37,38]. For this reason, we examined serum cystatin C levels and calculated the cystatin C eGFR in ALS as a way to gauge, independently from serum creatinine, their overall kidney function compared to HC. The results showed that there were no statistical differences in either serum cystatin C level or the calculated eGFR between ALS and HC. Additionally, there were no significant correlations found between either total or active MMP-9 and either cystatin C or eGFR. Thus, with regard to cystatin C and eGFR, there seemed to be no indication of significant kidney dysfunction among the ALS group nor any notable impact of kidney function on the levels of total and active MMP-9. However, we did observe both a significant positive correlation between the levels of serum NfL and cystatin C and a significant negative correlation between serum NfL and eGFR in ALS, but not in HC. This indicates that the kidney may have some effect on the serum levels of NfL in ALS.

Finally, we were interested in learning how the elevated levels of active MMP-9 observed in the serum of ALS compared with other inflammatory diseases, such as diabetic nephropathy, Alzheimer’s disease, and Parkinson’s disease. Because our study was primarily focused on ALS and because we had a limited amount of ALS samples, we only chose to look at a small sampling (n = 8) of patients diagnosed with those other diseases and compare them with an equal number of ALS and HC, with a priority of balancing all the groups for gender and age. We found that all the disease groups exhibited significantly higher mean levels of active MMP-9 as compared to HC. The ALS group (n = 8) had the highest mean levels of active MMP-9 of all of the groups, which was significantly higher than the Alzheimer’s (*p* = 0.0459), and HC (*p* < 0.0001) groups. As before, we were interested in knowing where these elevated active MMP-9 levels were originating from. First, we looked at muscle as a potential source through the analysis of total serum creatinine levels among the various disease groups. As expected, the nephropathy group exhibited significantly higher mean levels of serum creatinine than all of the other groups. The ALS group was found to have the lowest mean level of serum creatinine of all of the groups, which was significantly lower than the HC (*p* = 0.0345) and nephropathy groups (*p* = 0.0064), indicating again that the ALS group do not exhibit signs of renal dysfunction., We next examined the levels of serum cystatin C and calculated the eGFR for all of the disease groups. The nephropathy group exhibited the highest mean levels of cystatin C and lowest eGFR of all groups. However, because of the limited sample size, the only group showing significance was the calculated eGFR for Alz (*p* = 0.00721) with the other groups trending toward significance. We next looked at the levels of ALP between the various disease groups as a way to gauge for potential liver disease as another possible source for the elevated levels of total and active MMP-9. The results showed that there were no significant differences between any of the groups indicating that there is no indication of any significant liver problems and/or bone disorder.

The earlier results, showing no statistical differences in either serum cystatin C levels or the calculated eGFR between ALS and HC, indicate that the elevated serum levels of total and active MMP-9 are most likely not derived from the kidneys. However, more work is needed to definitively prove that the elevated levels originate from degenerating muscle. For example, future work could include a longitudinal study examining MMP-9 levels in both serum and CSF among ALS patients to determine whether the levels in the CSF approach those observed in serum. One could also investigate creatine kinase levels in serum to see if there is any correlation with the levels of total and active MMP-9, as a way to more directly gauge muscle function. Finally, access to biopsied muscle samples from ALS patients could provide critical information to determine whether total and active MMP-9 levels can be detected and are elevated in muscle tissue. Schoser and colleagues, using immunohistochemistry on muscle biopsies from a small cohort (n = 5) of patients with ALS, spinal muscular atrophy, and chronic axonal neuropathies, observed strong MMP-9 immunoreactivity—and to a lesser extent, MMP-2 and MMP-7—in all samples [39]. They also noted that the pattern of immunoreactivity differed markedly between the disease groups and normal muscle tissue. However, the method used did not allow for quantification or distinction between the pro- and active forms of the enzymes. Therefore, quantitative techniques would be necessary to definitively establish whether there is a link between total and active MMP-9 and degenerating muscle.

If the elevated levels of total and active MMP-9 are in fact derived from degenerating muscle, this may indicate that significant inflammatory processes are occurring peripherally to the CNS in ALS. Recently, there have been reports that phosphorylated TDP-43 (pTDP-43) aggregates have been detected in skeletal and cardiac muscle in ALS as well as in the GI tract and lymph nodes [40,41]. In some cases, these pTDP-43 aggregates are detected years before disease onset and formal ALS diagnosis [42]. The formation of these pTDP-43 aggregates outside of the CNS, and specifically in muscle, could be the initiator of these inflammatory processes and explain the upregulation of both total and active MMP-9 outside of the CNS. Given that a significant portion of the MMP-9 is in its active state and capable of cleaving proteins such as myelin basic protein, matrix proteins and activating various inflammatory cytokines, it is most probable that it is a significant source of further inflammatory activation, providing a compelling rationale for its inhibition not only in the CNS, but systemically, with an inhibitor such as AQU-118 as a potential approach to reducing ALS induced muscle degeneration.

## 4. Materials and Methods

Serum and CSF samples (Table 1). nVector provided the ALS serum samples and matched CSF samples (with only sex and age patient information available). The first group (Group 1, Table 1), consisting of serum samples from thirty people with ALS, was balanced for gender (18 males, 12 females) and age (mean ± SD, 59.4 ± 6.1 years). A second validation group (Group 3, Table 1), consisting of matching serum and CSF samples (Group 4, Table 1) from thirty people with ALS, was also balanced for gender (19 males, 11 females) and age (mean 60.9 ± 7.9 years).

Two sets of healthy age-matched controls (HC) were used in the study. One group of twenty serum samples from HC (Group 2, Table 1) was obtained from BioIVT and was balanced for gender (12 males, 8 females) and age (mean 56.5 ± 2.3 years). A second validation group of twenty serum samples from HC (Group 5, Table 1) was obtained from BioIVT and was balanced for gender (12 males, 8 females) and age (mean 60.6 ± 5.5 years). Fourteen CSF samples from people with other neurological diseases (OND) (Group 6, Table 1) was obtained from Precision for Medicine and were balanced for gender (8 males, 6 females) and age (mean 63.6 ± 9.8 years). The background information provided with samples included only the diagnosis or suspected diagnosis, the sex and age of the patients. This OND group included participants with optic neuritis, demyelinating disease of the CNS, idiopathic normal pressure hydrocephalus, acute cerebrovascular insufficiency, encephalopathy, hemiplegia, schizophrenia, and altered mental state.

Additionally, eight serum samples each (a total of 24 samples) were obtained from BioIVT from people diagnosed with diabetic nephropathy (Group, 9, 4 males, 4 females, 60.5 ± 6.8 years of age), Alzheimer’s disease (Group 10: 4 males, 4 females, 60.2 ± 5.5 years of age), and Parkinson’s disease (Group 11: 3 males, 5 females, 61.5 ± 2.9 years of age). The samples from diabetic nephropathy, Alzheimer’s disease and Parkinson’s disease were compared with an age-matched subset (Group 7: n = 8; 5 males, 3 females, 64.5 ± 8.4 years of age) taken from the second validation group of ALS serum samples and an age-matched subset (Group 8, n = 8; 4 males, 4 females, 61.1 ± 5.5 years of age) taken from the second validation group of HC serum samples.

The serum and CSF samples from ALS were obtained under a prior study protocol, which was ethically approved by an IRB (IRB#14BN059). The IRB approval included patient consent for the use of these samples for future research conducted by nVector. The HC samples obtained from BioIVT were collected under another study (Prospective Collection of Biological Specimens from Subjects Presenting at Specimen Donation Center for Research) by the commercial vendor, Seratrials/BioIVT, that was approved by an IRB (IRB#20161665) and included patient consent for the use of these samples for future research. The CSF samples from OND obtained from Precision for Medicine were collected under another study (PFM004, Remnant Biospecimen Program) by the commercial vendor, Precision for Medicine, that was approved by an IRB (IRB# Pro0051469) and included patient consent for the use of these samples for future research. All patient samples used in this study are legacy samples which included patient consent for their use in future research.

### 4.1. Chemical Materials

ProMMP-2 standard (Cat.444213-5 μg), active MMP-2 standard (Cat #PF023-5 μg), and ProMMP-9 standard (Cat #PF038-10 μg) were obtained from EMD Millipore (Burlington, VT, USA). 2× Zymogram Sample Buffer was obtained from G-BioSciences (St. Louis, MO, USA) (Cat # 786-483). Renaturing Buffer (Cat #LC2670) and Developing Buffer (Cat #LC2671) were Novex brand obtained from Thermo Fisher Scientific (Waltham, MA, USA). Simply Blue Stain was anInvitrogen brand obtained from Thermo Fisher Scientific (Waltham, MA, USA) (Cat #LC6060). Precision Plus Protein Dual Color Standards was obtained from Bio-Rad Laboratories (Waltham, MA, USA) (Cat. #161-0374). 10× TGS Running Buffer was obtained from Bio-Rad (Cat #161-0772). The electrophoresis tank was obtained from Invitrogen. The MMP inhibitor, AQU-118, was manufactured and purified by Aquilus Pharmaceuticals (Winchester, VA, USA) [26].

### 4.2. Measurement of Total MMP-2 and MMP-9 via ELISA

Total MMP-2 protein levels were determined in both serum and CSF using a commercially obtained enzyme-linked immunosorbent assay (ELISA) kit obtained from R & D Systems, Minneapolis, MN, USA (Total MMP-2 Quantikine ELISA Kit, MMP200). The assay was performed following manufacturer instructions included with the kit. Total MMP-9 protein was determined in both serum and CSF using ELISA obtained from R & D Systems (Human MMP-9 Quantikine ELISA kit, DMP 900). The assay was performed following manufacturer instructions included with the kit.

### 4.3. Measurement of Active MMP-2 via Antibody Capture and Colorimetric Assay

Active MMP-2 was measured using a commercially obtained Human MMP-2 Activity Assay v2.0 Kit (Quickzyme, Biosciences, Leiden, The Netherlands). The assay uses a precoated plate containing an antibody that can recognize both the active and pro forms of MMP-2. After the sample is exposed to the antibody, all of the non-MMP-2 proteins are washed away, leaving both the active and pro forms of MMP-2 attached. A detection enzyme is added. This detection enzyme is converted into an active form by any active MMP-2 present. The active detection enzyme cleaves a chromogenic substrate, resulting in the generation of a yellow color that can be measured at 405 nm. The assay was performed following manufacturer instructions included with the kit.

### 4.4. Measurement of Active MMP-9 via Antibody Capture and Fluorometric Assay

Active MMP-9 was measured using a commercially obtained Fluorokine E, Human Active MMP-9 Assay Kit (R & D Systems, Minneapolis, MN, USA, Cat#F9M00). The assay uses a precoated plate containing an antibody that can recognize both the active and pro forms of MMP-9. After the sample is exposed to the antibody, all of the non-MMP-9 proteins are washed away, leaving both the active and pro forms of MMP-9 attached. A FRET (fluorescence resonance energy transfer) peptide that is labeled with a fluorescence recorder is added to the bound enzymes. Only the active form can cleave the FRET peptide, which in turn generates fluorescence upon the peptide cleavage. The fluorescence measurement is related to the concentration of MMP-9 in the sample. The excitation wavelength is set to 320 nm and the emission wavelength is set to 405 nm. The assay was performed following manufacturer instructions included with the kit.

### 4.5. Measurement of Neurofilament Light (NfL) via ELISA

NfL protein was determined in both serum and CSF using a commercial ELISA kit obtained from MSD, Rockville, USA (S-PLEX Human Neurofilament L Kit). The assay was performed following manufacturer instructions included with the kit.

### 4.6. Measurement of Serum Creatinine

Creatinine was determined in serum using a commercially available colorimetric kit from Arbor Assays, Ann Arbor, MI, USA (Creatinine DetectX Colorimetric Detection Kit, Catalog #KB02-H). The assay was performed following manufacturer instructions included with the kit, which proposes a 30 min room temperature incubation prior to reading on a plate reader at 490 nm.

### 4.7. Measurement of Cystatin C via ELISA

Cystatin C was determined in serum using a commercially available ELISA kit from Arbor Assays (Human Cystatin C ELISA Kit, Cat. # K012-H1). The assay was performed following manufacturer instructions included with the kit.

### 4.8. Measurement of Alkaline Phosphatase

Alkaline phosphatase was determined in serum using a commercially available colorimetric kit from Arbor Assays (Alkaline Phosphatase Colorimetric Activity Kit, Cat. #K082-H1). The assay was performed following manufacturer instructions included with the kit, which proposes a 30 min incubation at 37 °C prior to reading on a plate reader at 405 nm.

### 4.9. Measurement of Inhibition of Active MMP-9 in Human Serum When Exposed to the MMP Inhibitor, AQU-118

AQU-118 is a proprietary, semi-selective MMP inhibitor capable of inhibiting both MMP-2 (IC50 = 2.0 nM) and MMP-9 (IC50 = 9 nM) and was synthesized by Aquilus Pharmaceuticals [26]. First, a 100 mg/ml solution of AQU-118 in triethylene glycol was prepared. The level of active MMP-9 was measured using the Fluorokine E, Human Active MMP-9 Assay Kit (R&D Systems, Minneapolis, MN, USA, Cat#F9M00) and a slightly modified procedure from that of the kit was used. Procedure: The samples from ALS were diluted to a final dilution of 1:100 into 1 × RD5-24 diluent containing a final concentration of inhibitor. Then, 50 microliters of Assay Diluent RD1N was added to each well of the plate. Then, 200 microliters of standard or sample was added to each well in duplicate. Each well was covered and shaken at 500 rpm for 2 h at room temperature. We washed 0/1/2/3 × with 300 microliters of 1× of Wash Buffer. We added 200 microliters of diluted substrate and incubated at 37 °C for 20 h. At the end of 20 h the Relative Fluorescence Unit (RFU) was measured using Ex/Em = 320/405 nm and 340/405 nm. The endpoint mode is present and the plate speed = 6.

### 4.10. Calculation of eGFR Levels

The eGFR was calculated using the method of Inker and colleagues [25] using the equation below:

eGFR = 133 × min(S_cys_/0.8, 1)^−0.499^ × max(S_cys_/0.8, 1)^−1.328^ × 0.996^Age^ × 0.932 [if female]

Abbreviations/Units

eGFR (estimated glomerular filtration rate) = mL/min/1.73 m^2^
S_cys_ (standardized serum cystatin C) = mg/L
min = indicates the minimum of S_cys_/0.8 or 1
max = indicates the maximum of S_cys_/0.8 or 1
age = years

### 4.11. Statistical Analysis

With regard to the two data sets of thirty ALS (Groups 1, 3 and 4, Table 1) and corresponding HC (Groups 2 and 5, Table 1) and ONC (Group 6, Table 1) groups, differences in means were compared by Welch’s *t*-test. Effect sizes were assessed with Cohen’s d. Correlations were assessed with both Pearson’s correlation and Spearman’s rank correlation. *p* values < 0.05 were considered significant. Prism version 8.02 (GraphPad Software, Boston, MS, USA) and R Studio 4.2.2 (developed by the R Foundation for Statistical Computing, https://www.r-project.org/about.html) were used for data visualization and analysis. With regard to the data sets between the different disease groups (Groups 7–11), comparisons were assessed by pairwise Welch’s *t*-test with Benjamini–Hockberg correction for multiple testing. All biomarker values are presented as mean ± standard deviation (SD).

## Figures and Tables

**Table 1:** Demographic Summary of Human Fluid Samples.

Group ID	Type of Sample	Disease	No. of Samples	No. of Males/Females	Mean Age ± SD (years)
1	Serum	ALS	30	18/12	59.4 ± 6.1 years
2	Serum	HC	20	12/8	56.5 ± 2.3 years
3	Serum (matched to CSF sample set #4)	ALS	30	19/11	60.9 ± 7.9 years
4	CSF (matched to serum sample set #3)	ALS	30	19/11	60.9 ± 7.9 years
5	Serum	HC	20	12/8	60.6 ± 5.5 years
6	CSF	OND	14	8/6	63.6 ± 9.8 years
7	Serum (subset from sample Set #3)	ALS	8	5/3	64.5 ± 8.4 years
8	Serum (subset from sample set #5	HC	8	4/4	61.1 ± 5.5 years
9	Serum	DN	8	4/4	60.5 ± 6.8 years
10	Serum	Alz	8	4/4	60.2 ± 5.5 years
11	Serum	Park	8	3/5	61.5 ± 2.9 years

ALS = Amyotrophic Lateral Sclerosis, Alz = Alzheimer's Disease, DN = Diabetic Nephropathy, HC = Healthy Control, OND = Other Neurological Diseases, Park = Parkinson's Disease.

## Data Availability

The data presented in this study are available upon request from the corresponding author (IS) due to intellectual property prosecution.

## Abbreviations

The following abbreviations are used in this manuscript:

ALP	Alkaline phosphatase
CSF	Cerebrospinal fluid
eGFR	Estimated glomerular filtration rate
ELISA	Enzyme-linked immunosorbent assay
FRET	Fluorescence resonance energy transfer
HC	Healthy controls
MMP-9	Matrix metalloproteinase 9
MMP-2	Matrix metalloproteinase 2
NfL	Neurofilament light
NGAL	Neutrophil gelatinase-associated lipocalin
OND	Other neurological diseases
TIMP	Tissue inhibitor of metalloproteinase

## References

[1] Bilana E., Boris V., Cena D., Veleska-Stefkovska D. (2011). Matrix metalloproteinases (with accent to collagenases). J. Cell Anim. Biol..

[2] Rodriguez D., Morrison C., Overall C.M. (2010). Matrix metalloproteinases: What do they not do? New substrates and biological roles identified by murine models and proteomics. Biochem. Biophys. Acta.

[3] Agrawal S.M., Lau L., Yong V.W. (2008). MMPs in the central nervous system: Where the good guys go bad. Semin. Cell Dev. Biol..

[4] Lee S.R., Lo E.H. (2004). Induction of caspase-mediated cell death by matrix metalloproteinase in cerebral endothelial cells after hypoxia-reoxygenation. J. Cereb. Blood Flow Metab..

[5] Beuche W., Yushchenko M., Mader M., Maliszewska M., Feigenhauer K., Wever F. (2000). Matrix metalloproteinase-9 is elevated in serum of patients with amyotrophic lateral sclerosis. Neuroreport.

[6] Lorenzl S., Albers D.S., LeWitt P.A., Chirichigna J.W., Hilgenberg S.L., Cudkowicz M.E., Beal M.F. (2003). Tissue inhibitors of matrix metalloproteinases are elevated in cerebrospinal fluid of neurodegenerative diseases. J. Neurol. Sci..

[7] Demestre M., Parkin-Smith G., Petzold A., Pullen A.H. (2005). The pro and the active form of matrix metalloproteinase-9 is increased in serum of patients with amyotrophic lateral sclerosis. J. Neuroimmunol..

[8] Niebroj-Dobosz I., Janik P., Sokolowska B., Kwiecinski H. (2010). Matrix metalloproteinases and their tissue inhibitors in serum and cerebrospinal fluid of patients with amyotrophic lateral sclerosis. Eur. J. Neurol..

[9] Fang L., Teuchert M., Huber-Abel F., Schattauer D., Hendrich C., Dorst J., Zettlmeissel H., Wlashek M., Scharffetter-Kochanek K., Kapfer T. (2010). MMP-2 and MMP-9 are elevated in spinal cord and skin in a mouse model of ALS. J. Neurol. Sci..

[10] Lukaszewicz-Zajac M., Mroczko B., Slowik A. (2014). Matrix metalloproteinase (MMPs) and their tissue inhibitors (TIMPs) in amyotrophic lateral sclerosis (ALS). J. Neural Transm..

[11] Huang F., Zhu Y., Hsiao-Nakamoto J., Tang X., Dugas J.C., Moscovitch-Lopatin M., Glass J.D., Brown R.H., Ladha S.S., Lacomis D. (2020). Longitudinal biomarkers in amyotrophic lateral sclerosis. Ann. Clin. Transl. Neurol..

[12] Thompson A.G., Gray E., Verber N., Bobeva Y., Lombardi V., Shepheard S.R., Yildiz O., Feneberg E., Farrimond L., Dharmadasa T. (2022). Multicentre appraisal of amyotrophic lateral sclerosis biofluid biomarkers shows primacy of blood neurofilament light chain. Brain Commun..

[13] Meyer T., Schumann P., Weydt P., Petri S., Koc Y., Spittel S., Bernsen S., Günther R., Weishaupt J.H., Dreger M. (2023). Neurofilament light-chain response during therapy with antisense oligonucleotide tofersen in SOD1-related ALS: Treatment experience in clinical practice. Muscle Nerve.

[14] Chiò A., Calvo A., Bovio G., Canosa A., Bertuzzo D., Galmozzi F., Cugnasco P., Clerico M., De Mercanti S., Bersano E. (2014). Piemonte and Valle d’Aosta Register for Amyotrophic Lateral Sclerosis. Amyotrophic lateral sclerosis outcome measures and the role of albumin and creatinine: A population-based study. JAMA Neurol..

[15] Cui C., Sun J., Pawitan Y., Piehl F., Chen H., Ingre C., Wirdefeldt K., Evans M., Andersson J., Carrero J.J. (2020). Creatinine and C-reactive protein in amyotrophic lateral sclerosis, multiple sclerosis and Parkinson’s disease. Brain Commun..

[16] Lombardi V., Querin G., Ziff O.J., Zampedri L., Martinelli I., Heller C., Foiani M., Bertolin C., Lu C.H., Malik B. (2019). Muscle and not neuronal biomarkers correlate with severity in spinal and bulbar muscular atrophy. Neurology.

[17] Wilson M.E., Boumaza I., Lacomis D., Bowser R., Feany M.B. (2010). Cystatin C: A candidate biomarker for amyotrophic lateral sclerosis. PLoS ONE.

[18] Zhu Y., Huo Y., Bai J., Li M., Wang H., Wang J., Huang X. (2023). Serum Cystatin C is a potential biomarker for predicting amyotrophic lateral sclerosis survival. Neurol. Sci..

[19] Rossano R., Larocca M., Macellaro M., Bilancia D., Riccio P. (2021). Unveiling a Hidden Biomarker of Inflammation and Tumor Progression: The 65 kDa Isoform of MMP-9 New Horizons for Therapy. Curr. Issues Mol. Biol..

[20] Olson M.W., Bernardo M.M., Pietila M., Gervasi D.C., Toth M., Kotra L.P., Massova I., Mobashery S., Fridman R. (2000). Characterization of the monomeric and dimeric forms of latent and active matrix metalloproteinase-9. Differential rates for activation by stromelysin 1. J. Biol. Chem..

[21] Vandooren J., Born B., Solomonov I., Zajac E., Saldova R., Senske M., Ugarte-Berzal E., Martens E., Van den Steen P.E., Van Damme J. (2015). Circular trimers of gelatinase B/matrix metalloproteinase-9 constitute a distinct population of functional enzyme molecules differentially regulated by tissue inhibitor of metalloproteinases-1. Biochem. J..

[22] Rossano R., Larocca M., Riviello L., Coniglio M.G., Vandooren J., Liuzzi G.M., Opdenakker G., Riccio P. (2013). Heterogeneity of serum gelatinases MMP-2 and MMP-9 isoforms and charge variants. J. Cell. Mol. Med..

[23] Petrozziello T., Mills A.N., Farhan S.M., Mueller K.A., Granucci E.J., Glajch K.E., Chan J., Chew S., Berry J.D., Sadri-Vakili G. (2020). Lipocalin-2 is increased in amyotrophic lateral sclerosis. Muscle Nerve.

[24] Brkic M., Balusu S., Libert C., Vandenbroucke R.E. (2015). Friends or foes: Matrix metalloproteinases and their multifaceted roles in neurodegenerative diseases. Mediat. Inflamm..

[25] Inker L.A., Schmid C.H., Tighiouart H., Eckfeldt J.H., Feldman H.I., Greene T., Kusek J.W., Manzi J., Van Lente F., Zhang Y.L. (2012). Estimating glomerular filtration rate from serum creatinine and cystatin C. N. Engl. J. Med..

[26] Kwan M.Y., Choo A., Hanania T., Ghavami A., Beltran J., Shea J., Barboza A., Hu A., Fowler M., Neelagiri V.R. (2019). Biomarker analysis of orally dosed, dual active, matrix metalloproteinase (MMP)-2 and MMP-9 inhibitor, AQU-118, in the spinal nerve ligation (SNL) rat model of neuropathic pain. Int. J. Mol. Sci..

[27] Ugarte-Berzal E., Martens E., Boon L., Vandooren J., Blockmans D., Proost P., Opdenakker G. (2018). EDTA/gelatin zymography method to identify C1s versus activated MMP-9 in plasma and immune complexes of patients with systemic lupus erythematosus. J. Cell. Mol. Med..

[28] Modvig S., Degn M., Horwitz H., Cramer S.P., Larsson H.B., Wanscher B., Sellebjerg F., Frederiksen J.L. (2013). Relationship between cerebrospinal fluid biomarkers for inflammation, demyelination and neurodegeneration in acute optic neuritis. PLoS ONE.

[29] Olsson B., Portelius E., Cullen N.C., Sandelius Å., Zetterberg H., Andreasson U., Höglund K., Irwin D.J., Grossman M., Weintraub D. (2019). Association of cerebrospinal fluid neurofilament light protein levels with cognition in patients with dementia, motor neuron disease, and movement disorders. JAMA Neurol..

[30] Steinruecke M., Lonergan R.M., Selvaraj B.T., Chandran S., Diaz-Castro B., Stavrou M. (2023). Blood-CNS barrier dysfunction in amyotrophic lateral sclerosis: Proposed mechanisms and clinical implications. Cereb. Blood Flow Metab..

[31] Chen X., Li Y. (2009). Role of matrix metalloproteinases in skeletal muscle: Migration, differentiation, regeneration and fibrosis. Cell Adhes. Migr..

[32] Li H., Mittal A., Paul P.K., Kumar M., Srivastava D.S., Tyagi S.C., Kumar A. (2009). Tumor necrosis factor-related weak inducer of apoptosis augments matrix metalloproteinase 9 (MMP-9) production in skeletal muscle through the activation of nuclear factor-κB-inducing kinase and p38 mitogen-activated protein kinase: A potential role of MMP-9 in myopathy. J. Biol. Chem..

[33] Ogura Y., Tajrishi M.M., Sato S., Hindi S.M., Kumar A. (2014). Therapeutic potential of matrix metalloproteinases in Duchenne muscular dystrophy. Front. Cell Dev. Biol..

[34] Alameddine H.S., Morgan J.E. (2016). Matrix Metalloproteinases and Tissue Inhibitor of Metalloproteinases in Inflammation and Fibrosis of Skeletal Muscles. J. Neuromuscul. Dis..

[35] Baeza-Trinidad R., Brea-Hernando A., Morera-Rodriguez S., Brito-Diaz Y., Sanchez-Hernandez S., El Bikri L., Ramalle-Gomara E., Garcia-Alvarez J.L. (2015). Creatinine as predictor value of mortality and acute kidney injury in rhabdomyolysis. Intern. Med. J..

[36] Nagel G., Kurz D., Peter R.S., Rosenbohm A., Koenig W., Dupuis L., Bäzner H., Börtlein A., Dempewolf S., Schabet M. (2023). Cystatin C based estimation of chronic kidney disease and amyotrophic lateral sclerosis in the ALS registry Swabia: Associated risk and prognostic value. Sci. Rep..

[37] Rodríguez-Sánchez E., Navarro-García J.A., Aceves-Ripoll J., Abarca-Zabalía J., Susmozas-Sánchez A., Bada-Bosch T., Hernández E., Mérida-Herrero E., Andrés A., Praga M. (2020). Variations in circulating active MMP-9 levels during renal replacement therapy. Biomolecules.

[38] Tashiro K., Koyanagi I., Ohara I., Ito T., Saitoh A., Horikoshi S., Tomino Y. (2004). Levels of urinary matrix metalloproteinase-9 (MMP-9) and renal injuries in patients with type 2 diabetic nephropathy. J. Clin. Lab. Anal..

[39] Schoser B.G.H., Blottner D. (1999). Matrix metalloproteinases MMP-2, MMP-7 and MMP-9 in denervated human muscle. NeuroReport.

[40] Cykowski M.D., Powell S.Z., Appel J.W., Arumanayagam A.S., Rivera A.L., Appel S.H. (2018). Phosphorylated TDP-43 (pTDP-43) aggregates in the axial skeletal muscle of patients with sporadic and familial amyotrophic lateral sclerosis. Acta Neuropathol. Commun..

[41] Mori F., Tada M., Kon T., Miki Y., Tanji K., Kurotaki H., Tomiyama M., Ishihara T., Onodera O., Kakita A. (2019). Phosphorylated TDP-43 aggregates in skeletal and cardiac muscle are a marker of myogenic degeneration in amyotrophic lateral sclerosis and various conditions. Acta Neuropathol. Commun..

[42] Pattle S.B., O’SHaughnessy J., Kantelberg O., Rifai O.M., Pate J., Nellany K., Hays N., Arends M.J., Horrocks M.H., Waldron F.M. (2022). pTDP-43 aggregates accumulate in non-central nervous system tissues prior to symptom onset in amyotrophic lateral sclerosis: A case series linking archival surgical biopsies with clinical phenotypic data. J. Pathol. Clin. Res..

